# Study protocol. TRAAP - TRAnexamic Acid for Preventing postpartum hemorrhage after vaginal delivery: a multicenter randomized, double-blind, placebo-controlled trial

**DOI:** 10.1186/s12884-015-0573-5

**Published:** 2015-06-14

**Authors:** Loïc Sentilhes, Valérie Daniel, Astrid Darsonval, Philippe Deruelle, Delphine Vardon, Franck Perrotin, Camille Le Ray, Marie-Victoire Senat, Norbert Winer, Françoise Maillard, Catherine Deneux-Tharaux

**Affiliations:** Department of Obstetrics and Gynecology, Angers University Hospital, 4, rue Larrey, 49933 Angers, France; Department of Pharmacy, Angers University Hospital, Angers, France; PPRIGO (Production Pharmaceutique pour la Recherche Institutionnelle du Grand Ouest) Brest University Hospital, Brest, France; Department of Obstetrics and Gynecology, Jeanne de Flandre University Hospital, Lille, France; Department of Obstetrics and Gynecology, Caen University Hospital, Caen, France; Department of Obstetrics and Gynecology, Tours University Hospital, Tours, France; Port-Royal Maternity Unit, Department of Obstetrics and Gynecology, Cochin University Hospital, APHP, Paris, France; INSERM, Obstetrical, Perinatal and Pediatric Epidemiology Research Team, Center for Epidemiology and Biostatistics (U1153), Risks in pregnancy DHU, Paris-Descartes University, Paris, France; Department of Obstetrics and Gynecology, Kremlin-Bicetre University Hospital, APHP, Paris, France; Department of Obstetrics and Gynecology, Nantes University Hospital, Nantes, France

**Keywords:** Postpartum hemorrhage, Treatment, Prevention, Tranexamic acid, Cesarean and vaginal deliveries, Thrombosis

## Abstract

**Background:**

Postpartum hemorrhage (PPH) is a major cause of maternal mortality, accounting for one quarter of all maternal deaths worldwide. Estimates of its incidence in the literature vary widely, from 3 % to 15 % of deliveries. Uterotonics after birth are the only intervention that has been shown to be effective in preventing PPH. Tranexamic acid (TXA), an antifibrinolytic agent, has been investigated as a potentially useful complement to uterotonics for prevention because it has been proved to reduce blood loss in elective surgery, bleeding in trauma patients, and menstrual blood loss. Randomized controlled trials for PPH prevention after cesarean (*n* = 10) and vaginal (*n* = 2) deliveries show that women who received TXA had significantly less postpartum blood loss without any increase in their rate of severe adverse effects. However, the quality of these trials was poor and they were not designed to test the effect of TXA on the reduction of PPH incidence. Large, adequately powered, multicenter randomized controlled trials are required before the widespread use of TXA to prevent PPH can be recommended.

**Methods and design:**

A multicenter, double-blind, randomized controlled trial will be performed. It will involve 4000 women in labor for a planned vaginal singleton delivery, at a term ≥ 35 weeks. Treatment (either TXA 1 g or placebo) will be administered intravenously just after birth. Prophylactic oxytocin will be administered to all women. The primary outcome will be the incidence of PPH, defined by blood loss ≥500 mL, measured with a graduated collector bag. This study will have 80 % power to show a 30 % reduction in the incidence of PPH, from 10.0 % to 7.0 %.

**Discussion:**

In addition to prophylactic uterotonic administration, a complementary component of the management of third stage of labor acting on the coagulation process may be useful in preventing PPH. TXA is a promising candidate drug, inexpensive, easy to administer, and simple to add to the routine management of deliveries in hospitals. This large, adequately powered, multicenter, randomized placebo-controlled trial seeks to determine if the risk-benefit ratio favors the routine use of TXA after delivery to prevent PPH.

**Trial registration:**

ClinicalTrials.gov NCT02302456 (November 17, 2014)

## Background

### Rational

#### Active management of the third stage of labor for the prevention of postpartum hemorrhage

Postpartum hemorrhage (PPH) is classically defined as blood loss of 500 mL or more after delivery [1]. Estimates of its incidence in the literature vary widely, from 3 % to 15 % of deliveries [2–5]. About one in five of these hemorrhages progresses to a severe form that may endanger the mother’s life or at least her future fertility and exposes her to the risks of transfusion, surgery, and intensive care. PPH remains a leading cause of maternal mortality and accounts for about one quarter of all maternal deaths worldwide. Its risk factors include previous PPH, primiparity, obesity, prolonged or augmented labor, multiple pregnancy, previous cesarean delivery, polyhydramnios, and macrosomia [4, 5]. Nevertheless, most women with PPH have low-risk pregnancies and no identifiable risk factors [6, 7]. It is therefore essential to prevent PPH in all women [8–11].

Because placental expulsion is a critical window for the prevention of PPH, various preventive interventions during this stage have been proposed. They can be schematically divided into two categories: those involving a mechanical mechanism, and those involving prohemostatic agents. Active management of the third stage of labor (AMTSL) comprises a combination of mechanical interventions: preventive administration of uterotonic agents immediately after delivery of the child, early cord clamping and cutting, controlled cord traction (CCT), and, according to some authors, uterine massage [12]. Nevertheless, it has been clearly shown that the administration of uterotonics, and in particular oxytocin, after birth is the only component of AMTSL that is effective in preventing PPH [13–17]. However, in addition to this enhancement of mechanical hemostasis, a complementary biochemical hemostatic effect might be expected from the complementary use of prohemostatic drugs such as tranexamic acid.

#### Tranexamic acid for the reduction of bleeding in medical contexts other than PPH

Tranexamic acid (TXA) is a potent antifibrinolytic agent that exerts its effect by blocking lysine binding sites on plasminogen molecules and has the potential to enhance the effectiveness of the patient’s own hemostatic mechanisms. Consequently, clot breakdown (fibrinolysis) is inhibited and bleeding is reduced [18]. Results from previous trials have shown that TXA in planned surgery reduces the risk of blood transfusion, mean transfused volume, and need for re-operation due to bleeding, without increasing thrombotic events [19, 20]. The CRASH-2 trial (Clinical Randomisation of an Antifibrinolytic in Significant Haemorrhage) demonstrated that the administration of TXA reduced mortality in bleeding trauma patients in high-, middle-, and low-income countries [21]. Significant reductions in mean menstrual blood loss have been reported in women with menorrhagia treated with TXA, compared with control or placebo-treated women [22]. This result is particularly significant since the efficacy of TXA in menorrhagia suggests that it can reduce uterine blood loss, even of low volume, and in a nonsurgical context [23].

#### Tranexamic acid for PPH

During delivery, when the placenta separates from the uterine wall, sequential physiologic and hemostatic changes occur and reduce bleeding, including strong myometrial contractions, increased platelet activity, and a massive release of coagulant factors; at the same time, however, fibrinolytic activity increases [24]. While oxytocin administration enhances the first mechanism, TXA administration might be able to counter the latter and thus facilitate the hemostatic process. Finally, the close relation observed between reduced fibrinogen levels and outcome in cases of PPH [25, 26] further suggests that TXA might be effective in PPH [27].

##### Treatment of PPH

The only randomized controlled trial (multicenter open-label trial) to assess TXA for the treatment of PPH showed that a high dose of TXA reduces blood loss in women with PPH [27]. Due to its limitations (small sample size, open design, issues regarding the harm-benefit ratio), and conflicting results from a before-and-after study using the same protocol [28], the use of TXA to treat PPH remains the object of debate. A pragmatic, randomized, double-blinded, placebo-controlled trial in women with a clinical diagnosis of PPH, the WOMAN trial, has been launched to rectify this clinical uncertainty [29]. It is currently recruiting eligible women in hospitals in Africa and Europe, aiming to reach the target sample size of 15 000 women [29].

##### Prevention of PPH

A Cochrane systematic review published in 2011 examined the use of TXA to prevent PPH. It identified 5 RCTs and included 2 of them in the meta-analysis, which covered a total of 453 women [18]. The authors concluded that the available evidence, although it suggested that TXA decreases postpartum blood loss, was of unclear quality and that further investigation was needed. Several additional trials have been published since then.

Through a Medline search up to March 1, 2014, we identified 12 RCTs that have assessed this issue (Tables 1 and 2) [30–41]. All were performed in low-or middle-income countries (China, India, Iran, Turkey, Pakistan, and Egypt). All but two [40, 41] only included women with cesarean deliveries. Overall, the quality of these trials was poor. Selection, performance, and detection bias might have influenced their results; in particular, the primary outcome was never the incidence of either PPH or severe PPH but rather other indicators of blood loss that may not be clinically relevant. The method used to measure the primary outcome based on estimated blood loss was often imprecise and subject to both performance and detection bias. These flaws mandate a cautious interpretation of their results [18, 42].

**Table 1:** Characteristics of the randomized trials that have assessed tranexamic acid for preventing postpartum hemorrhage after cesarean delivery

Study [réf]	Country	Study design	Sample size	Study groups	Intervention	TXA Dosage/route	Primary outcome	Result	P value	Adverse effects
Gai et al. al, (2004) [30]	China	Multicenter, prospective, randomized controlled study	N = 180, primiparas	N = 91 (experimental)	Infusion of TXA 10 min before CS	1 g IV for 5 min	Postpartum blood loss not clearly specified	359.3 mL vs 439.3 mL	0.002	No thromboembolic or other side effects reported
				N = 89 (no placebo)						
Gohel et al., (2007) [31]	India	Prospective, randomized controlled study	N = 100, primiparas and multiparas	N = 50 (experimental)	Infusion of TXA 20 min before CS	1 g IV for 5 min	Postpartum blood loss not clearly specified	374.9 mL vs 472.8 mL	0.003	No thromboembolic or other side effects reported
				N = 50 (no placebo)						
Sekhavat et al., (2009) [32]	Iran	Prospective, randomized, placebo-controlled study	N = 90, primiparas	N = 45 (experimental)	Infusion of TXA 10 min before CS	1 g IV for 5 min	Postpartum blood loss not clearly specific	28.0 mL vs 37.1 mL	0.001	No thromboembolic or other side effects reported
				N = 45 (placebo)						
Gungorkuk et al., (2011) [33]	Turkey	Prospective, unicenter, double-blind, randomised controlled study	N = 666, primiparas and multiparas	N = 330 (experimental)	Infusion of TXA 10 min before CS	1 g IV for 5 min	Estimated blood loss during CS.	600.7 mL vs 499.9 mL	<0.001	Gastrointestinal side effects (16.3 %) in the experimental group
				N = 330 (placebo)						
										Gastrointestinal side effects not mentioned for the placebo group.
										No thromboembolic events
Movafegh et al., 2011 [34]	Iran	Prospective, unicenter, double-blind, randomized controlled study	N = 100, primiparas and multiparas	N = 50 (experimental)	Infusion of TXA 20 min before CS	10 mg/kg IV for 10 min	Postpartum blood loss not clearly specified	262.5 mL vs 404.7 mL	<0.001	No thromboembolic events
				N = 50 (placebo)						
Xu et al., 2013 [35]	China	Randomized, double-blind, placebo-controlled study	N = 174 primiparas	N = 88 (experimental)	Infusion of TXA 10 min before CS	10 mg/kg IV for 5 min	Postpartum blood loss not clearly specified	379 mL vs 441 mL	0.02	2 thromboses occurred in each group.
										Gastrointestinal side effects occurred in 10 TXA patients versus one placebo patient
				N = 86 (placebo)						
Senturk et al., 2013 [36]	Turkey	Randomized, double-blind, placebo-controlled study	N = 223, primiparas and multiparas	N = 101 (experimental)	Infusion of TXA 10 min before CS	10 mg/kg IV for 5 min	Postpartum blood loss not clearly specified	272 mL vs 347 mL	0.001	No thromboembolic or gastrointestinal side effects
				N = 122 (placebo)						
Shahid A et al., 2013 [37]	Pakistan	Randomized double-blind placebo controlled study	N = 74 primiparas and multiparas	N = 38 (experimental)	Infusion of TXA 10 min before CS	1 g IV for 10 min	Postpartum blood loss not clearly specified	356 mL vs 710 mL	<0.001	No thromboembolic side effects
				N = 36 (placebo)						
Abdel-Aleem et al., 2013 [38]	Egypt	Randomized, single center, open, controlled study	N = 740 primiparas and multiparas	N = 373 (experimental)	Infusion of TXA 10 min before CS	1 g IV for 10 min	Blood loss two hours after delivery	241.6 mL vs 510.6 mL	<0.001	Gastrointestinal side effects (74.3 % versus 53.1 %; *P* = 0.0001)
				N = 367 (no placebo)						
										No thromboembolic side effects
Goswami et al., 2013 [39]	India	Randomized, single center, double-blinded placebo controlled study	N = 90 primiparas and multiparas	N = 30 (experimental 1)	Infusion of TXA 20 min before CS	Experimental 1: 10 mg/kg	Postpartum blood loss, not clearly specified	376.8 mL vs 261.2 mL vs 527.2 mL	Not reported	No thromboembolic side effects
				N = 30 (experimental 2)						
						Experimental 2: 15 mg/kg				
				N = 30 (placebo)						

*TXA* Tranexamic acid, *IV* intravenous

**Table 2:** Characteristics of the randomized trials that have assessed tranexamic acid for preventing postpartum hemorrhage after vaginal delivery

Study [réf]	Country	Study design	Sample size	Study groups	Interventions	TXA Dosage/route	Primary outcome	Result	P value	Adverse effects
Yang et al., (2001) [40]	China	Multicenter, randomized controlled study	N = 400 primiparas	N = 94 (experimental 1)	Infusion of TXA after delivery of the fetal shoulders in the first 2 groups and infusion of AMBA in the third	1 g IV.	Measurement of blood loss 2 h after delivery, without details about the measurement method	243.3 mL vs 242.9 mL vs 308.1 mL vs 314.8 mL	<0.01	Nausea (n = 2)
						0.5 g IV				
				N = 92 (experimental 2)						
				N = 92 (experimental 3)		0.5 g IV				
				N = 87 (no placebo)		placebo				
Gungorkuk et al., (2013) [41]	Turkey	Prospective, single-center, double-blinded, randomized controlled study	N = 439 primiparas and multiparas	N = 220 (experimental) N = 219 (placebo)	Infusion of TXA at delivery of the anterior shoulder	1 g IV for 5 min	Mean blood loss during the third and fourth stages of labor - from the end of delivery to 2 h postpartum- measured as (weight of material used – weight of materials before use)/1.05	261.5 mL vs 349.9 mL	<0.001	Gastrointestinal side effects (35.9 %)
										No thromboembolic event

*TXA* Tranexamic acid, *AMBA* aminomethylbenzoic acid, *IV* intravenous

We found 10 published RCTs evaluating the efficacy of TXA in preventing PPH after cesarean delivery [30–39]. Their characteristics are summarized in Table 1. In all but one of these studies [36], the cesareans were elective. These 10 RCTs all reported a significantly smaller mean postpartum blood loss in women who had received TXA, and no detectable adverse effect on their primary vital signs (blood pressure, heart rate, and respiratory rate) or thrombosis (Table 1) [30–39]. Nevertheless, it is important to emphasize that most of these RCTs have various methodological flaws and included small samples with inadequate power to assess the risk of adverse effects.

Only 2 trials evaluated the efficacy of TXA for the prevention of PPH after vaginal delivery [40, 41] (Table 2). In 2001, Yang al. reported a RCT comparing four groups [26]. One group (*n* = 94) received a single dose of 1 g TXA by intravenous infusion (IV), and another group (*n* = 92) a single dose of 0.5 g TXA, also IV. The third group (n =92) received a single dose of 0.5 g aminomethylbenzoic acid IV, and the fourth group (*n* = 87) served as the control group [26]. Defining PPH as blood loss ≥ 400 mL, the authors reported that its incidence was lower in the women receiving the larger TXA dose (6/94) than the control group (22/87) (Table 2). The pooled relative risk of PPH was thus RR = 0.44 (95 % CI: 0.31 to 0.64) [26, 34]. The main limitation of this study is the absence of placebo and blinding. Moreover, the randomization method was not described.

More recently, Gundorkuk et al. recruited 439 women with vaginal deliveries in a double-blinded RCT [41] (Table 2). Women in the intervention group received a single dose of 1.0 g of TXA IV at delivery of the anterior shoulder, and those in the control group a placebo. The primary outcome was the mean estimated blood loss during the third and fourth stages of labor. It was significantly lower in the TXA group than in the placebo group (261.5 ± 146.8 mL compared with 349.98 ± 188.85 mL, *P* <0.001). The incidence of PPH >500 mL was also lower in the TXA group (*n* = 4, 1.8 %) than in the control group (*n* = 15, 6.8 %); RR 3.76; 95 % CI: 1.27 to 1.15, *P* = 0.01) [41]. Moreover, the rate of total blood loss of at least 1000 mL was higher among women who received placebo (2.3 %) than among women given TXA (0.5 %), but the difference was not statistically significant (RR 5.02, CI: 0.59 to 42.64, *P* = 0.12). The groups did not differ significantly in their need for blood transfusion. Predelivery hemoglobin and hematocrit levels were the same in both groups, but the day after delivery, both hemoglobin (9.9 ± 1.4 g/dL and 9.3 ± 0.9 g/dL, *P* < 0.001) and hematocrit levels (30.2 % ± 1.2 % and 29.0 ± 1.3, *P* < 0.001) were higher in the TXA than the placebo group [41]. Finally, no episodes of thrombosis occurred in the women who received TXA. The results of this single-center trial suggest that TXA is a promising drug for the prevention of PPH after vaginal delivery and possibly for the reduction of maternal morbidity related to PPH. Nevertheless, there are again some concerns about the external validity of these results observed in a middle-income country, partly because it was a single-center study and, relatedly, because it was underpowered to assess severe adverse events.

### Severe maternal adverse events related to tranexamic acid

Caution is necessary before recommending the use of this drug in routine practice for prevention or treatment of PPH, in view of the number of eligible women who would be eligible to receive it in both cases: not only is the evidence supporting its efficacy still insufficient, but also and especially the number of venous and arterial thrombotic events might increase after its administration. Consequently, until now, no authorities or learned societies have recommended the administration of TXA to prevent PPH, after either vaginal or cesarean delivery.

Because TXA inhibits fibrinolysis, it carries a potential risk of thrombosis, especially in patients with a previous history of thrombosis and in pregnant women. Nevertheless, the systematic review of randomized controlled trials of elective surgical patients cited above (129 randomized controlled trials including 10 488 randomized participants, 5484 of them allocated to TXA) showed no significant increases in the incidence of myocardial infarction, stroke, deep vein thrombosis, or pulmonary embolism [20]. Moreover, the CRASH-2 trial of TXA in bleeding trauma patients showed a statistically significant reduction in global mortality with no increase in thromboembolic events [21]. Nevertheless, as thromboembolic events are relatively rare, these trials do not resolve the uncertainties about this risk because most of these trials lacked the statistical power to detect clinically important increases in risk. In particular, five cases of acute renal failure due to thrombosis-induced cortical necrosis following TXA administration have been described [43–47].

The evidence available for assessing the safety of TXA use in parturients for prevention of PPH is even more limited than for nonpregnant patients, given the limited number of such women thus far enrolled in TXA trials and the expected incidence of these adverse events. It is well known that the risk of venous thromboembolism increases during normal pregnancy and especially during the first 6 weeks postpartum [48]. It is nonetheless important to underline that no significant increase in the incidence of thrombotic events related to TXA has been observed in any trials of TXA among pregnant women (*n* = 2228), including RCTs, observational nonrandomized studies and case series [49].

In conclusion, TXA appears to be a promising drug for the prevention of PPH, but the results currently available are too limited to justify its widespread use. Given the current uncertainty regarding both its efficacy and its adverse effects in parturient women, we propose to perform an adequately powered, multicenter, randomized, placebo-controlled trial to determine the impact of systematic TXA administration after vaginal delivery on PPH incidence. If TXA is effective, its routine use should reduce the incidence of PPH among women with vaginal deliveries. If TXA is shown to be ineffective, this will prevent the diffusion of an ineffective medication associated with potential adverse effects in routine practice.

The hypothesis is that a low dose of TXA (1 g) after vaginal delivery, administered in the 2 min after the child’s delivery and after prophylactic oxytocin administration, reduces both blood loss and the incidence of PPH and its complications.

### Objectives

The aim of this study is to compare the effect of a low dose of TXA (1 g) after vaginal delivery in the 2 min after the child’s delivery and after prophylactic oxytocin administration, versus placebo with prophylactic oxytocin in a multicenter randomized, double-blind, placebo-controlled trial.

The specific objectives are as follows:

- To assess the impact of TXA (1 g) after vaginal delivery on postpartum blood loss
  - ■ Primary outcome
    - ➢Incidence of PPH, defined by blood loss ≥ 500 mL, measured with a graduated collector bag
  - ■ Secondary outcomes
    - ➢Other outcome measures describing postpartum blood loss, such as mean measured total postpartum blood loss, incidence of severe PPH, defined by blood loss ≥ 1000 mL, proportion of women requiring supplementary uterotonic agent, transfusion, arterial embolization, or emergency surgery for PPH, and mean peripartum change in hemoglobin and hematocrit
- To assess the potential adverse effects of TXA (1 g) after vaginal delivery:
  - ■ The hemodynamic parameters, gastrointestinal side effects, renal, hepatic and coagulation function, venous or arterial thrombosis within 3 months following delivery.
- To assess women’s satisfaction and psychological status at D60

## Methods and design

### Recruitment and allocation

#### Inclusion criteria

- Age ≥ 18 years
- Planned vaginal delivery
- Term ≥ 35 weeks of gestation
- Singleton pregnancy
- Informed consent form signed

#### Exclusion criteria

- History of venous (deep vein thrombosis and/or pulmonary embolism) or arterial (angina pectoris, myocardial infarction, stroke) thrombosis
- History of epilepsy or seizure
- Any known cardiovascular, renal, or liver disorders
- Autoimmune disease
- Sickle cell disease
- Severe hemorrhagic disease
- Placenta previa
- Abnormally invasive placenta (placenta accreta/increta/percreta)
- Abruptio placentae
- Eclampsia, HELLP syndrome
- Multiple pregnancy
- In utero fetal death
- Administration of low-molecular-weight heparin or antiplatelet agents during the week before delivery
- Poor understanding of the French language

Individual information on the trial will be provided to women in late pregnancy by obstetricians and midwives during prenatal visits or by anesthetists during the systematic anesthesia visit, or both. This information will be repeated when the women arrive in the delivery room; each woman will then confirm her participation and provide informed written consent before delivery, when, in the investigator’s opinion once she has reached at least 4 cm of cervical dilatation, she is likely to have a vaginal delivery.

The randomization will be centralized and stratified by center and balanced in blocks of variable sizes. Once a woman has been included, through the filing of an electronic case report directly by internet (Clinsight software), she will retain her randomization number (if it has been assigned to her) even if she withdraws from the study or refuses randomization afterwards.

Women will be randomized to receive either 1 g of TXA (Sanofi Aventis, Paris, France; Marketing authorization number: 3400931157618 [1974, RCP rév 02.04.2010]) or placebo (normal saline, Fresenius Kabi, Sèvres, France; Marketing authorization number: 34009415 73941). The Angers Clinical Research Unit will create the randomization list for allocations to the TXA and control groups. This list will be transmitted to the pharmacy department of CHU Angers, which belongs to the PPRIGO hospital pharmacists’ consortium (Production Pharmaceutique pour la Recherche Institutionnelle du Grand Ouest). The blinded products will be prepared by the Angers CHU pharmacy according to GMP for hospital pharmacies (BPP 2007), which will provide numbered and labeled boxes, each containing a 10-mL vial of the study drug (1 g of TXA or placebo according to the randomization number). All boxes and vials will be identically labeled, with the study number being the only differentiating feature between different drug packs. This will ensure the woman’s safety and the blinding of all participants, including obstetric staff.

### Intervention

The intervention will be the intravenous administration of a 10-mL blinded vial of the study drug (either 1 g of TXA or placebo, according to the randomization group), slowly (over 30–60 sec), in the 2 min after birth and the routine prophylactic oxytocin, and after the cord has been clamped, all generally by the midwife (but sometimes by the anesthesiologist or obstetrician, depending on availability).

Except for the content of the study drug vial, all aspects of management of the third stage will be identical in both arms:

- Routine prophylactic intravenous injection of 5 IU oxytocin at delivery of the anterior shoulder or within 2 min of birth, as recommended in the national clinical practice guidelines issued by the French College of Gynecologists and Obstetricians [50].
- Placement of a graduated (100 mL graduation) collector bag just after birth, left in place for at least 15 min and then until the birth attendant judges that bleeding has stopped. When a woman is included in the trial, a bag will be prepared and ready to be put in place as soon as the baby is born and placed on the mother’s belly; if needed, a second staff person will be present to help in managing both the baby and the bag. This will make it possible to collect and measure vaginal blood loss objectively during the immediate postpartum [14].
- Manual removal of the placenta at 30 min after birth if not expelled, in the absence of bleeding.
- Rapid suturing of the episiotomy, in accordance with good clinical practices.
- Systematic use of uterotonic drugs after the third stage of labor is not recommended.
- Controlled cord traction (CCT) will be left to the discretion of the practitioner since its use in the management of placental expulsion has been shown to have no significant effect on the incidence of PPH or other markers of postpartum blood loss [14].

If PPH occurs, standardized management will be provided according to the department’s protocol. In particular, the use of TXA for the treatment of PPH will be allowed and left to the discretion of the practitioner, according to the department’s protocol.

Before inclusions begin, a meeting will be organized in each maternity unit to verify the attendants’ agreement to and understanding of the protocol and their ease in practicing the relevant procedures.

### Outcome measures

#### Primary outcome measure

The primary outcome of the trial is the incidence of PPH, defined by blood loss ≥ 500 mL, measured with a graduated collector bag. The term “measured” implies an objective determination of the blood volume lost, in contrast to a simple visual estimate, which has been shown unreliable in several studies [14, 51–55].

The systematic use of a graduated collector bag for blood, placed under the woman’s buttocks just after delivery of the child, will allow an objective measurement of the blood lost through the vagina in the immediate postpartum period.

The practical feasibility of the use of such a bag was shown in the TRACOR trial [14]. This bag shall be left in place for at least 15 min and afterwards until the birth attendant is no longer concerned about blood loss, and judges that the woman’s condition allows her to stretch out. This criterion will be measured during the immediate postpartum surveillance in the delivery room, in both groups.

#### Secondary outcomes

- Secondary outcome measures describing postpartum blood loss

#### Clinical

- mean measured blood loss at 15 min after birth (the collector bag must be left in place at least 15 min to have one measure of blood loss at the same time point for all women)
- mean measured total postpartum blood loss (at bag removal)
- incidence of severe PPH, defined by measured blood loss ≥ 1000 mL.
- proportion of women requiring supplementary uterotonic treatment, including sulprostone
- incidence of postpartum transfusion (until discharge)
- incidence of arterial embolization and emergency surgery for PPH.

#### Laboratory

- mean change in peripartum hemoglobin (difference between Hb before delivery and at D2)
- mean change in peripartum hematocrit (difference between Ht before delivery and on D2).

For both indicators, the pre-delivery reference examination will be the last routine blood count in the last trimester of the pregnancy. A blood sample will be taken from all women included in the trial on the second day postpartum for the measurement of peripartum Hb and Ht and the calculation of the change in these two indicators. If no blood sample is available from D2, measurement of postpartum Hb and Ht will be assessed from a D3 blood sample, if available. If no blood sample is available from D2 or D3, measurement of postpartum Hb and Ht will be assessed from a D1 blood sample, if available.

- The occurrence of potential adverse effects of TXA:

#### Clinical

- Hemodynamic parameters (heart rate, blood pressure) 15, 30, 45, 60 and 120 min after delivery.
- the occurrence of potential mild adverse effects of TXA in the delivery room:
  - ■ nausea
  - ■ vomiting
  - ■ phosphenes
  - ■ dizziness
- the occurrence of potential severe adverse effects of TXA up to three months of postpartum:

- ■ deep vein thrombosis, if the diagnosis is confirmed by Doppler ultrasound
- ■ pulmonary embolism, if the diagnosis is confirmed by radiological examinations.
- ■ myocardial infarction
- ■ seizure
- ■ renal failure needing dialysis
- ■ any other unexpected adverse events.

These events will be checked by the medical team during the hospitalization and then on D90 by a telephone interview of each woman. In the event that the woman cannot be reached, at least 10 calls at different hours over the course of the week will be made to minimize loss to follow-up. In cases of severe adverse effects reported by a woman after the hospital discharge, objective data will be collected from medical files, transmitted either by the woman herself or her general practitioner.

#### Laboratory

- urea and creatinemia, prothrombin time (PT), active prothrombin time (aPTT), aspartate and alanine transaminase, and total bilirubin at D2

- Women’s satisfaction and psychological status

- These will be assessed by a self-administered questionnaire on day 2 postpartum and by mail on day 60.

### Statistical analysis

Data analysis and reporting will follow the CONSORT guidelines for randomized controlled trials and will be conducted with the trial statistician and researchers blinded to group status. The two groups will be compared for women’s demographic characteristics and standard risk factors for PPH. Then we will seek, in an intention-to-treat analysis, the existence of a “TXA effect”, that is, a difference between the two groups for the primary outcome measure and the secondary outcome measures. For secondary outcomes that are available for women who had a cesarean delivery after randomization (percentage of women requiring supplementary uterotonic agents, mean change in peripartum hemoglobin, mean change in peripartum hematocrit, incidence of postpartum transfusion, arterial embolization, and emergency surgery), a secondary analysis will be conducted that includes these women. The comparisons will use nonparametric statistical tests when required by the constraints of population size or distributions. Comparisons of the categorical variables will use chi2 and Fisher exact tests, as appropriate. We will use analysis of variance and Kruskal and Wallis’s nonparametric test for the continuous variables. Effects will be measured as relative risks, with their 95 % confidence intervals.

The analysis plan includes an intermediate statistical analysis when half of the planned women have been included to ensure there is no significant difference in the adverse events between the two groups.

### Feasibility

The majority of the participating centers have previously worked together in a French multicenter randomized controlled trial that aimed to assess the effect of controlled traction of the cord on the prevention of PPH (TRACOR trial) [17]. During a one-year period, they together randomized 4068 women with vaginal deliveries and demonstrated their capacity to conduct and perform a RCT regarding the prevention of PPH. Moreover, all the participating centers belong to the GROG (Groupe de Recherche en Obstétrique et Gynécologie) national network.

### Sample size

In the recent RCT (TRACOR trial), involving the same participating centers, mentioned above, comparison of the efficacy of the controlled cord traction and standard placenta expulsion in preventing PPH showed that the PPH rate (measured postpartum blood loss of 500 mL or more) was 10.0 % (402/4013), with no significant heterogeneity between centers [14].

Accordingly, for this study, we assumed a 10 % incidence of PPH in the absence of TXA. To show a relative reduction of at least 30 % in this incidence in the TXA arm—that is, an incidence of 7 % or less in this arm, with a = 0.05, 1-ß = 0.90, and a bilateral test, the study requires 1814 women with vaginal deliveries in each group. Given the expected proportion of women with a cesarean delivery in labor after randomization (estimated at 5 % to 10 % [6.8 % in the TRACOR trial] [14]), the total number of randomized women will be greater in order to include the needed number of women with vaginal deliveries: 2000 women will be included in each group, for a total of 4000 participants.

### Timetable

The participating maternity units deliver 21,500 infants a year. We consider that 70 % of women will meet the criteria for inclusion in the participating maternity units, excluding scheduled cesareans, cesareans in early labor, deliveries before 35 weeks, and multiple pregnancies. We add to these categories the cesareans decided at the end of labor (about 5 % of deliveries), after inclusion but for which no primary outcome measure will be available, and which therefore cannot be included in the analysis — even for intention to treat. Overall, 13,975 annual deliveries (65 % of the total) may possibly be included in the principal analysis. An inclusion period of 20 months should make it possible to recruit 4,000 women, if we assume a participation rate of at least 17.2 %. This participation rate appears realistic in light of the recruitment in the TRACOR trial in the labor wards of all the participating centers [17].

The total duration of the trial will be 23 months, including 20 months of inclusion and 3 months of follow-up in the postpartum period (assessment of thromboembolic and any unexpected adverse events).

The total duration of the project will be 34 months, including:

- 6 months for ethics approval, signatures of agreements between the participating centers and training the staff in the management methods required by the protocol for the third stage of labor.
- 23 months for the trial, with an inclusion period of 16 months and a follow-up period of 3 months.
- 5 months after the end of the inclusion period will be devoted to finalizing the data collection and entry, database cleaning, and analysis.

### Ethics committee approval

This study was approved by the West II Committee for the Protection of Research Subjects (Ethics Committee) on August 21, 2014 (2014/09), and by the French Health Products Safety Agency (ANSM) on August 6, 2014 (2014-001748-39).

### Data management

Each investigator will be responsible for ensuring data, which will be completed by the clinical research technician throughout the trial, with Clinsight software. The electronic case report file for each woman will contain 5 components:

- 1 completed by the clinical research technician from the obstetric file: woman’s characteristics, course of the pregnancy, labor, and delivery
- 1 completed by the clinical research technician about the postpartum events after leaving the delivery room, and the results of the blood count on D2
- 1 questionnaire about women’s satisfaction on D2 postpartum, completed by the women, with responses entered secondarily in the electronic file by the clinical research technician.
- 1 questionnaire about the women’s satisfaction and psychological status on D60 postpartum, sent by the technician to the women and completed by them, with responses entered secondarily in the electronic file by the clinical research technician.
- 1 questionnaire about the occurrence of thromboembolic and any other unexpected events at 12 weeks in postpartum, completed by the clinical research technician during a telephone interview with the woman, with responses secondarily entered in the electronic file by the technician.

Data will be collected for women who have a cesarean delivery after randomization, data will be collected.

The data management and statistical aspects will be handled centrally by the Angers University Hospital (Department of Clinical Research and Development - Methodology and Biostatistics Unit) with the Scientific Director of the study (CDT).

Quality control will be conducted according to the standard operating procedures of the sponsor, Angers University Hospital. The research in the investigational centers and management of subjects will comply with the Declaration of Helsinki and Good Clinical Practices. An independent data monitoring committee including only independent members will be in charge of controlling the quality and the safety of the trial procedures and of the data collected. Clinical research assistants will conduct monthly visits in each investigative center and report to the data monitoring committee. During these onsite inspections and in accordance with Good Clinical Practices, the following items will be reviewed:

- Compliance with the research protocol and the procedures defined therein
- Verification of the patients’ informed consents, for all women included
- Examination of the source documents and their comparison with the data reported in the electronic case report files (CRFs), for the accuracy and consistency of the data and the missing data, performed on a random sample of 10 % of participating women.
- End-of-trial visit: archiving of research documents.

### Trial steering committee

A trial steering committee will be set up which will have overall supervision of the trial. It will meet before the trial starts and then at least every 6 months until completion. A meeting of the TSC will be held within a month of every Safety Monitoring Committee meeting to consider their recommendations.

### Safety consideration

An independent Safety Monitoring Committee will also be formed. They will meet at minimum yearly to examine recruitment figures, baseline data, retention and adverse events. The Safety Monitoring Committee will review the result of the interim analysis of safety data when half of the planned participants have been recruited.

Suspected unexpected serious adverse reactions (SUSARs) will be recorded and reported using the ANSM approved SUSAR form. SUSARs include maternal death, surgery (other than cesarean delivery) in the 12 weeks following randomization, transfusion of more than 4 units of blood, admission to an intensive care unit, deep vein thrombosis, pulmonary embolism, myocardial infarction, seizure, renal failure defined by the need for dialysis, or suspected drug reactions. In the event of a SUSAR the form will be completed by the local trial coordinator and faxed to the trial coordinating center at the Angers University Hospital within 72 h. From there the copies of the form will be sent to the trial statistician and the chair of the Safety Monitoring Committee. The Safety Monitoring Committee will have the power to ask to break the blinding, whereas the study investigators will remain blinded to the allocated treatment. The ANSM, the trial sponsor and the Chair of the Ethics Committee will also be informed by the Safety Monitoring Committee if considered appropriate, in particular in cases of strong suspicion of SUSARS related to the Investigational Medicinal Products. Finally, should the Safety Monitoring Committee believe it necessary, it is authorized to recommend, to the Scientific Committee that the trial be stopped.

## Discussion

### Potential and implementation of the findings

In addition to prophylactic uterotonic administration, a complementary component of the management of third stage of labor that acts on the coagulation process may be useful for PPH prevention. This prevention is especially important as several reports from various high-resource countries describe recent increases in PPH incidence [3, 56, 57]. TXA is a promising candidate drug, inexpensive and easy to administer, that could be added to the routine delivery management of all women in hospitals worldwide. The evidence currently available is too limited to justify its widespread use for PPH prevention. This large, adequately powered, multicenter, randomized placebo-controlled trial aims to determine if the risk-benefit ratio favors the systematic use of TXA after delivery to prevent PPH.

### Dissemination

A final report will be prepared for the funding body, and papers will be prepared for peer-review publication and national/international dissemination.

## Conclusion

Both theoretical arguments and results from RCTs conducted in other clinical contexts indicate that TXA has promise in the prevention of PPH. Nevertheless, the available evidence from RCTs is of insufficient quality to reach any definitive conclusion, although it does suggest that TXA administration reduces postpartum blood loss. We hope that the issue of TXA for the prevention of PPH following a vaginal delivery will be informed by the results of this large, adequately powered, multicenter, randomized placebo-controlled trial.
